# Genome-wide acquired uniparental disomy as well as chromosomal gains and losses in an uterine epithelioid leiomyoma

**DOI:** 10.1186/1755-8166-7-19

**Published:** 2014-03-03

**Authors:** Carsten Holzmann, Dominique Nadine Markowski, Dirk Koczan, Burkhard Maria Helmke, Jörn Bullerdiek

**Affiliations:** 1 Institute for Medical Genetics, University of Rostock, University Medicine, Ernst-Heydemann-Strasse 8, D-18057 Rostock, Germany; 2 Center of Human Genetics, University of Bremen, Leobener Strasse ZHG, D-28359 Bremen, Germany; 3 Institute for Immunology, University of Rostock, University Medicine, Schillingallee 70, D-18057 Rostock, Germany; 4 Institute of Pathology, University of Heidelberg, Heidelberg, Germany; 5 Present address of B.M.H.: Institute of Pathology, Elbe Kliniken, Klinikum Stade, Bremervörder Str. 111, D- 21682 Stade, Germany

**Keywords:** Uterine leiomyoma, Genetics, Uniparental disomy, Loss of heterozygosity, Haploid karyotype

## Abstract

**Background:**

Epitheloid leiomyoma is a rare subtype of benign smooth muscle tumors.

**Results:**

Herein, we present the results of classical cytogenetics, *MED12* mutation analysis, and copy number variation array evaluation in one such case. Whereas cytogenetic did not show evidence for clonal chromosome abnormalities and no *MED12* mutation in the “fibroid hot spot” region was detected, array hybridization revealed multiple abnormalities. Most noteworthy, almost all chromosomes showed copy-number neutral loss of heterozygosity. As examples of further abnormalities, trisomies of chromosomes 8, 12, 20, and X were noted.

**Discussion:**

The data presented suggest a near-haploid karyotype of the tumor as the initial genetic alteration followed by secondary duplications of large parts of the genome. The absence of any clonal karyotypic alterations after performing classical cytogenetics is likely explained by a reduced ability of the tumor cells to proliferate in vitro. However, to the best of our knowledge this is the first report of an uterine leiomyoma showing extended uniparental disomy. It remains to be determined if this is a more common phenomenon in epithelioid leiomyomas or even subsets of “ordinary” leiomyomas.

## Background

Epithelioid leiomyoma is a rare variant of uterine leiomyomas (UL) accounting for less than 1% of these tumors. Nevertheless, clinical presentation as well as its histology suggests a considerable degree of heterogeneity among these lesions. Benign uterine smooth muscle tumors neither carrying clonal cytogenetic deviations nor mutations of the gene encoding mediator subcomplex 12 (*MED12*) [1], as predominantly found in cytogenetically normal UL [2], are rare. In an attempt to identify genetic alterations in these lesions, we have analyzed an epithelioid UL by copy number variation (CNV)-array in addition to karyotyping and *MED12* sequencing. By the two latter methods the tumor did neither display clonal karyotypic deviations nor a *MED12* mutation akin to those found in a large percentage of UL [1]. Besides numerical aberrations of chromosomes 8, 12, 20, and X and some small gains and losses that had all escaped detection by classical cytogenetics, the tumor showed uniparental disomy for most of the chromosomes except for those affected by trisomies and tetrasomies. The pattern of genetic alterations clearly distinguishes this lesion from ordinary UL.

## Case presentation

**Figure 1:**
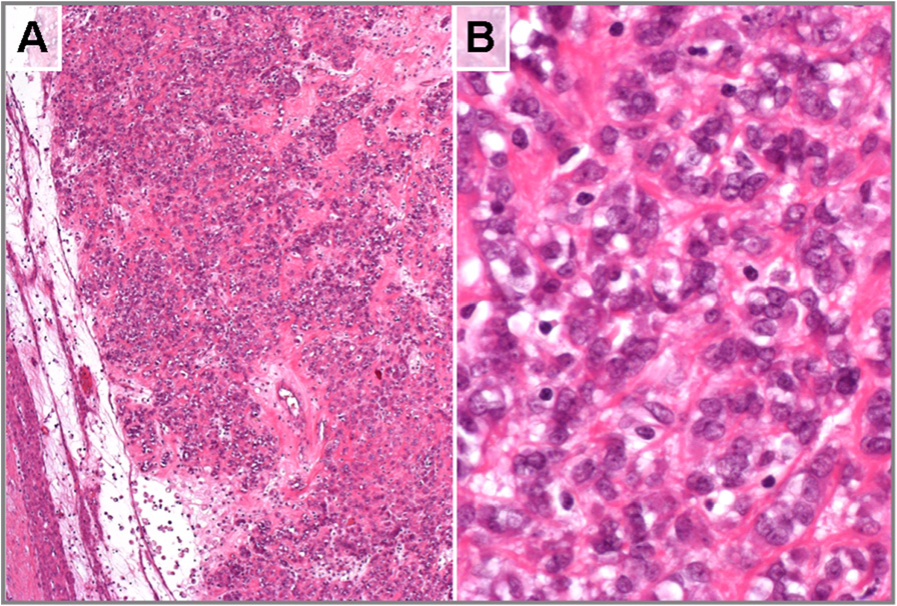
**Histologic appearance of a so-called epithelioid uterine smooth muscle tumor.** Showing nests of cells displaying an epithelial-like morphology embedded in a partly myxoid stroma. **(A)**. At a higher magnification part of the cells present with prominent nuclei **(B)**.

**Figure 2:**
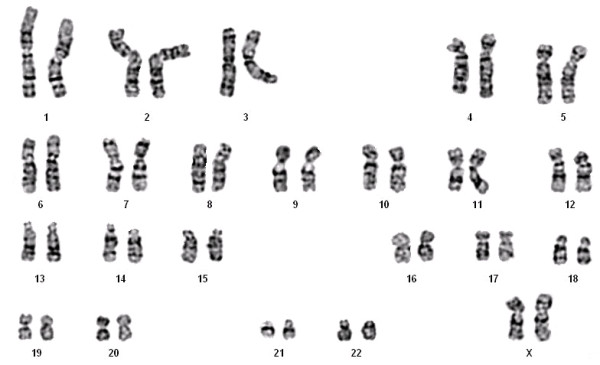
**Representative G-banded karyotype.** Displaying no numerical or structural karyotype deviations.

A 45 year old patient was admitted to the hospital because of a large intramural uterine tumor which was removed by hysterectomy. As a gross finding, a 7 cm well-circumscribed tumor was found. Histologically, the tumor presented as an encapsulated so-called epithelioid leiomyoma (Figure 1) with mild nuclear atypica but no enhanced mitotic activity or necrotic areas. There were no signs of local invasiveness. Besides this single nodule no further uterine tumors were detected. By classical cytogenetics, no clonal karyptypic abnormalities were detected among a total of 20 metaphases fully analyzed (band resolution: 300 -500bands per haploid set) (Figure 2) but one of the metaphases examined showed a 45, XX, -8, +12, -15 karyotype.

In addition to cytogenetic examination, the tumor was also analyzed for *MED12* mutations by DNA-sequencing as described earlier [1]. As a result, the tumor did not display a *MED12* mutation as commonly found in UL (data not shown).

**Figure 3:**
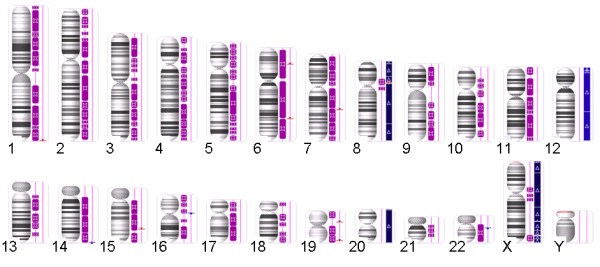
**Karyotype of the tumor visualized in Affymetrix Chromosome Analysis Suite v2.0.1 [ChAS] software**. Long contiguous stretches of homozygosity are (purple bars) observed in most chromosomes. The copy number state segments are shown in red (losses) or blue bars (gains, light blue for copy number 3, dark blue for copy number 4).

Finally, the tumor was investigated by whole genome copy number variation (CNV)-arrays. Despite its apparently normal karyotype in cell culture, this latter analysis detected various genetic abnormalities. Most remarkably, the tumor displayed large areas and even whole chromosomes with copy-number neutral loss of heterozygosity (Figure 3).

**Figure 4:**
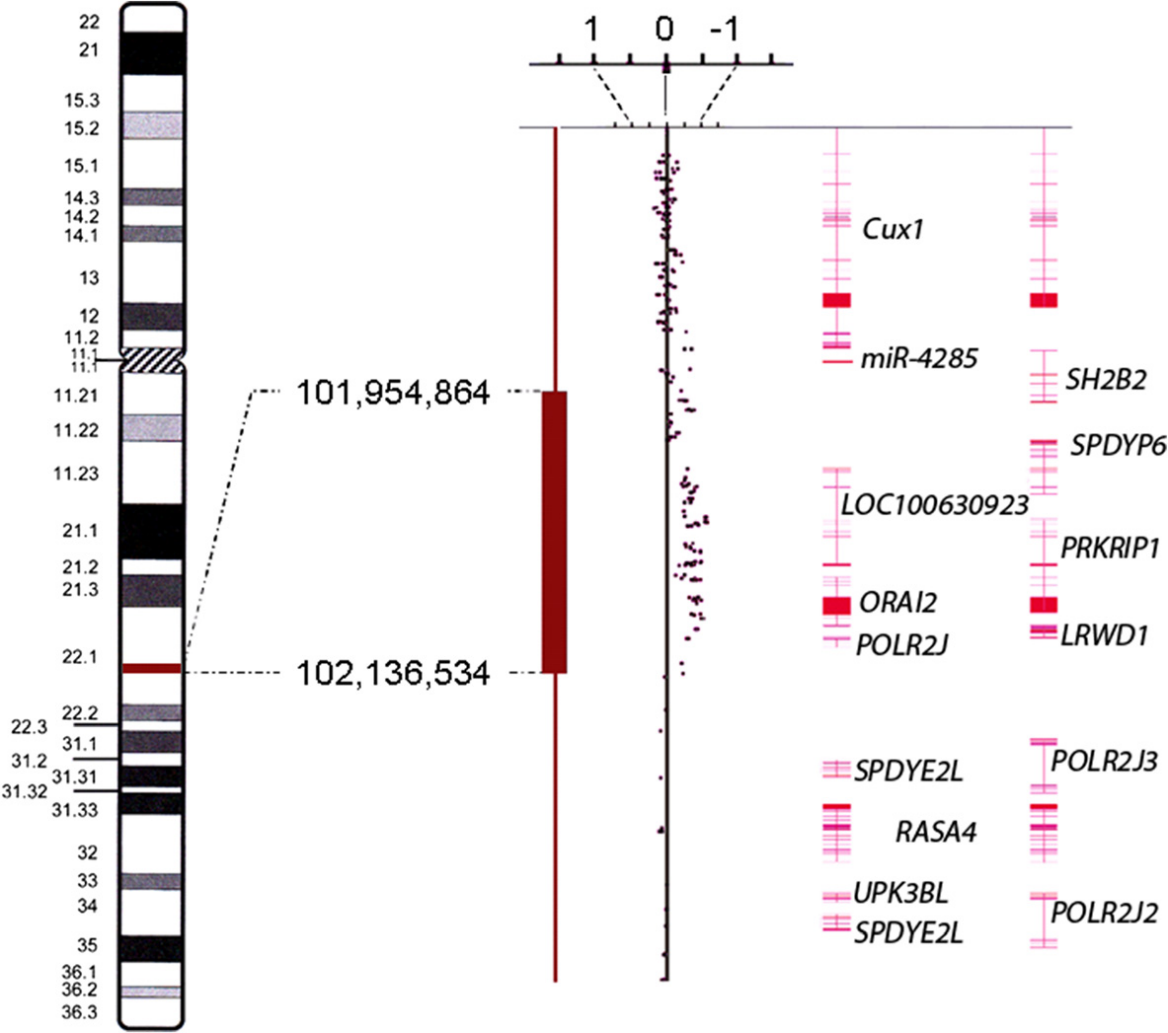
**Detail view of chromosome 7. **Copy number state segment (red bar for deletion) and copy number state data of a part of chromosomal sub-band 7q22.1. Each dot represents the copy number state (weighted log2 ratio) of one marker. The right part of the figure shows the exon-intron-structure of the genes in the displayed region. Numbering of positions is based on hg19 (NCBI Build 37 reference sequence).

Of note, only chromosomes 8, 12, 20, and the X-chromosome lacked this uniparental disomy but these chromosomes showed copy number gains with three (chromosome 12) or three to four copies (chromosomes 8, 20, and X) each. Chromosome 8 and the X-chromosome presented with these copy number gains as well as with large discontinuous stretches of uniparental disomy in a few other chromosomes, small deletions were seen within otherwise long chromosomal regions of homozygosity (Table 1). Of these, one affecting a small segment on the long arm of chromosome 7 (Figure 4) deserves particular interest because it lies within the common region of overlap of cytogenetically visible deletions of 7q. As the target gene of these deletions *CUX1* has been proposed earlier [3-5].

**Table 1:** Genetic abnormalities detected by whole genome CNV + SNP array hybridization

**Chromosome**	**Type**	**CN**	**Cytobands**	**Size (kbp)**	**Genes**
1	UPD	2	All		
	Loss	1	q44	74.216	OR2T2, OR2T3, OR2T5
2	UPD	2	All		
3	UPD	2	All		
4	UPD	2	All		
5	UPD	2	All		
6	UPD	2	All		
	Loss	1	p22.1	71.464	HCG4B, HLA-A
	Loss	1	q22.33	189.413	TMEM244, L3MBTL3
7	UPD	2	All		
	Loss	1	q22.1	181.67	SH2B2, SPDYE6, LOC100289561, LOC100630923, PRKRIP1, ORAI2, ALKBH4, LRWD1, MIR5090, MIR4467, POLR2J
8	Gain	4	All		
	LOH	4	p12–p11.1	11659.74	71 genes
	LOH	4	q23.3–q24.3	6392.362	14 genes
	Loss	0	p11.22	139.855	ADAM5P, ADAM3A
9	UPD	2	All		
10	UPD	2	All		
11	UPD	2	All		
12	Gain	3	All		
13	UPD	2	All		
14	UPD	2	All		
	Gain	3	q32.33	324.063	KIAA0125, ADAM6
15	UPD	2	All		
	Loss	1	q24.3	40.641	SCAPER
16	UPD	2	All		
17	UPD	2	All		
18	UPD	2	All		
19	UPD	2	All		
	Loss	1	p12	677.28	ZNF682, ZNF90, ZNF486, MIR1270-2, MIR1270-1, ZNF826P, ZNF737
	Loss	1	q13.42	107.4	KIR2DL3, KIR2DL1, LOC100287534, KIR2DL4, KIR3DL1, KIR2DS4
20	Gain	4	All		
21	UPD	2	All		
22	UPD	2	All		
	Gain	3	q11.22	138.446	MIR650, IGLL5
X	Gain	4	All		
	LOH	4	p11.23–p11.1	8936.629	88 genes
	LOH	4	q11.1–q13.1	5950.032	20 genes
	LOH	4	q13.1–q21.1	5907.594	58 genes
	LOH	2	q27.2–q27.3	1422261	3 genes
	LOH	4	q27.3–q28	10557350	90 genes

*Abbreviations: CN*, Copy number.

However, for none of the genetic alterations the evaluation offered evidence for a significant proportion of cells not carrying these alterations and its restriction to a subset of the tumor cell population has been obtained.

## Conclusions

In the case presented herein, array-based CNV detection not only allowed to detect copy number variations but also copy-number-neutral loss of heterozygosity (LOH) or uniparental disomy, respectively. Apparently, most chromosomes were in a disomic stated while they had lost heterozygosity. The large extension of this copy-number-neutral LOH [6] allows for the conclusion that it has developed as a somatic alteration. As the most likely explanation, the alterations detected have arisen from a near-haploid tumor retaining e.g. both chromosomes 12 with secondary duplication of the chromosome complement. The occurrence of near haploid karyotypes has been observed in a variety of solid tumors including leiomyosarcomas [7] as well as in leukemias [8]. Also, copy-number-neutral LOH affecting more or less large segments has been observed in many cancers [9]. When it affects larger parts of the genome, secondary duplications have been proposed as the underlying mechanism. Duplication of the chromosomes as a later event has e.g. been suggested to explain findings obtained by array hybridization on chondrosarcomas. In a study of sixteen chondrosarcomas that had been investigated by single nucleotide polymorphism (SNP) arrays, the majority of the tumors displayed SNP patterns indicative of a hyperhaploid-hypodiploid origin, with or without subsequent polyploidization [10]. Despite chromosomal gains detected by array analysis in the present case, no clonal chromosome abnormalities were found by classical cytogenetics. The small deletion of 7q which was noted coincides with a commonly deleted region in UL [3-5], but is too small to be detected by classical cytogenetics. The proximal border of the deleted segment lies in close proximity of *CUX1* but does not affect that gene. In contrast, this proximal breakpoint is located within *SH2B2*. Nevertheless, the deletion may have removed downstream regulating sequences of *CUX1* thus not excluding *CUX1* as a possible target.

As to the molecular pathogenesis of the tumor, this deletion as well as the gain of chromosome 12 are frequent findings in other UL too and are likely to have contributed to tumor development either as primary or secondary alterations besides UPD.

In contrast, another explanation may account for the lack of numerical chromosome alterations as clonal karyotype deviations. Recent findings of an in vitro selection against tumor cells in some subtypes of fibroids [11] suggest that this absence reflects an in vitro artefact.

In summary, to the best of our knowledge this is the first report of an uterine leiomyoma showing extended uniparental disomy. It remains to be determined if it is a more common phenomenon in epithelioid leiomyomas or even in subsets of ordinary leiomyomas.

## Methods

### Histologic examination

The tumor was fixed in paraformaldehyde (4% in PBS) and processed for paraffin embedding. Tissue sections (1–2 μm thickness) were deparaffinized in xylene, rehydrated through a series of ethanol, and stained with hematoxylin and eosin (H&E).

### Cytogenetic studies

Chromosome analyses of cell cultures were performed following routine techniques as described earlier [12].

### DNA isolation

DNA from the frozen tissue sample was isolated using the QIAamp DNA Mini Kit (Qiagen, Hilden, Germany) on a QIACube (Qiagen) according to the manufacturer’s instructions.

### PCR and sequencing

For PCR amplification 1000 ng of genomic template DNA were used. Primers to amplify the desired human PCR fragment of the *MED12* gene were those recently described [1,2]. Subsequently, PCR-products were separated by agarose gel-electrophoresis and the desired DNA-fragments/-bands were extracted by a QIAquick Gel Extraction Kit (Qiagen) using a QIACube (Qiagen) according to manufacturer’s instructions. DNA-sequencing of the purified PCR-products was performed by GATC Biotech (Konstanz, Germany).

### Arrays

CNV (copy number variation) analysis was performed using premade CytoScan HD Arrays (Affymetrix, Santa Clara, CA) consisting of more than 2.4 million markers for copy number and approximately 750,000 single nucleotide polymorphisms (SNPs). Enriched gene coverage for cancer and constitutional genes results in marker-base ratio cover ages of 1/384 for ISCA, 1/553 for cancer genes, 1/486 for X-chromosomal genes and 1/659 for 12,000 OMIM genes. Labelling of 250 ng DNA and hybridization were done following the manufacturer’s instructions. After staining and washing using a GeneChip Fluidics Station 450 (Affymetrix) the arrays were scanned by an Affymetrix 3000 7G scanner. Arrays were analyzed through the Affymetrix Chromosome Analysis Suite (ChAS) software (ChAS analysis files for CytoScan® HD Array version NA32.3). Numbering of map positions was based on hg19 (NCBI Build 37 reference sequence).

## Consent

The study was approved by the local ethics committee (Ethikkomission bei der Ärztekammer Bremen). Samples were obtained in accordance with the declaration of Helsinki and prior to surgery, informed written consent was obtained from the patient. A copy of the written consent is available for review by the Editor-in-Chief of this journal.

## Competing interests

The authors declare that they have no competing interests.

## Authors’ contributions

CH: conception and design of the study; acquisition of data; analysis and interpretation of data; manuscript writing; final approval of the manuscript. DNM: conception and design of the study; acquisition of data; analysis and interpretation of data; manuscript writing; final approval of the manuscript. DK: acquisition of data; analysis and interpretation of data; final approval of the manuscript. BMH: analysis and interpretation of data; provision of study material; final approval of the manuscript. JB: conception and design of the study; analysis and interpretation of data; manuscript writing; revising the manuscript critically for important intellectual content; final approval of manuscript.
